# The Endothelial Dysfunction Blocker ameliorates cognitive impairment by inhibiting blood‐brain barrier (BBB) dysfunction and neuroinflammation in the 5XFAD mouse model of Alzheimer's disease

**DOI:** 10.1002/alz70855_100124

**Published:** 2025-12-26

**Authors:** Young‐Guen Kwon, Cho‐Rong Bae, Yeomyeong Kim, Dongyeop Kim

**Affiliations:** ^1^ Curacle Co. Ltd, Seoul, Seoul, Korea, Republic of (South)

## Abstract

**Background:**

Blood‐brain barrier (BBB) dysfunction and neuroinflammation play a crucial role in the pathogenesis of Alzheiemr's disease (AD). Recent studies have reported that preserving or restoring blood‐brain barrier (BBB) function could be a promising strategy for mitigating AD progression and severity. Our endothelial dysfunction blocker, CU71, enhanced tight junction and vascular integrity while decreased inflammation in human brain microvascular endothelial cell (HBMECs). We investigated the effects of CU71 on cognitive impairment BBB dysfunction, and neuroinflammatory activity in the 5XFAD mouse model of Alzheimer's disease.

**Method:**

5XFAD mice received daily oral administration of CU71 for 6 months. Cognitive functions were assessed using Y‐maze, novel object recognition (NOR) and Morris water maze (MWM) tests. The study included 5 groups: (1) wild type (WT)+vehicle, (2) 5XFAD (TG)+vehicle, (3) TG +Donepezil (1 mg/kg), (4) TG+5 mg/kg CU71, (5) TG+10 mg/kg CU71. To examine the molecular mechanisms related to BBB dysfunction and neuroinflammation, immunohistochemistry, western blot and digital ELISA assay (SIMOA) were performed.

**Result:**

After 6 months of CU71 treatment, significant improvements in memory and cognition were observed, as indicated by enhanced such as NOR and MWM tests, compared to the TG + vehicle treatment control group. Additionally, CU71 treatment improved vessel maintenance and rescued tight junction‐related proteins. We also observed a decrease in the expression of glial fibrillary acidic protein (GFAP) and ionized calcium‐binding adapter molecule 1 (Iba‐1). Therefore, CU71 may be useful for treating cognitive dysfunctions observed in AD.

**Conclusion:**

Our findings demonstrate that CU71 treatment ameliorates cognitive impairment by inhibiting BBB dysfunction and neuroinflammation in the 5XFAD mouse model of Alzheimer's disease

